# Supplementary material for: Adverse Childhood Events Significantly Impact Depression and Mental Distress in Adults with a History of Cancer
Source: Cancers (Basel). 2024 Sep 27;16(19):3290. doi: 10.3390/cancers16193290 (PMC11476032; doi:10.3390/cancers16193290)
Supplement: Supplementary file 1 [file cancers-16-03290-s001.zip › cancers-3146480-supplementary.pdf]

Supplemental Table S1. Granular age and race/ethnicity distribution.

	N (%)
**Age at time of survey**	
18-39	352 (5.4)
40-64	3850 (34.5)
≥65	9712 (60.1)
**Race/ethnicity**	
Non-Hispanic White	12660 (81.5)
Non-Hispanic Black	349 (4.5)
Hispanic	330 (6.6)
Non-Hispanic Other	793 (7.4)

Supplemental Table S2. Multivariable logistic regression model estimating association between adverse childhood experience and depression using granular age and race/ethnicity.

	**aOR (95% CI)**
# of ACEs	
Zero	Reference
1-2	1.84 (1.47, 2.31)
≥3	3.73 (2.89, 4.81)
Missing	2.08 (1.55, 2.80)
**Age at survey**	
18-39	2.87 (1.80, 4.56)
40-64	1.68 (1.40, 2.02)
≥65	Reference
**Race/ethnicity**	
Non-Hispanic White	Reference
Non-Hispanic Black	0.74 (0.43, 1.25)
Hispanic	0.73 (0.47, 1.14)
Non-Hispanic Other	1.68 (1.17, 2.42)

Model adjusted for age at survey, gender, race/ethnicity, marital status, education level, income level, body mass index, smoking status, and health status.

Supplemental Table S3. Multinomial regression examining association between adverse childhood experience and mental distress, 2022 Behavioral Risk Factor Surveillance System (n=14,132).

	aOR (95% CI)	
	**No vs. Yes mental distress**	**Missing vs. Yes mental distress**
# of ACEs		
Zero	Reference	Reference
1-2	0.99 (0.72, 1.36)	0.70 (0.54, 0.92)
≥3	0.67 (0.48, 0.93)	0.30 (0.23, 0.40)
Missing	0.96 (0.65, 1.43)	0.59 (0.42, 0.82)

Model adjusted for age at survey, gender, race/ethnicity, marital status, education level, income level, body mass index, smoking status, and health status.
